# Nod-like Receptors: Critical Intracellular Sensors for Host Protection and Cell Death in Microbial and Parasitic Infections

**DOI:** 10.3390/ijms222111398

**Published:** 2021-10-22

**Authors:** Abdulkareem Olarewaju Babamale, Szu-Ting Chen

**Affiliations:** 1 Taiwan International Graduate Program in Molecular Medicine, National Yang-Ming Chiao Tung University and Academia Sinica, Taipei 11266, Taiwan; babamale.oa@unilorin.edu.ng; 2 Parasitology Unit, Faculty of Life Sciences, University of Ilorin, Ilorin 240003, Nigeria; 3 Institute of Clinical Medicine, National Yang-Ming Chiao Tung University, Taipei 11266, Taiwan; 4 Cancer Progression Research Center, National Yang-Ming Chiao Tung University, Taipei 11266, Taiwan

**Keywords:** NLRP12, cell death, inflammasome, IFN-I, caspase 1, NF-κB

## Abstract

Cell death is an essential immunological apparatus of host defense, but dysregulation of mutually inclusive cell deaths poses severe threats during microbial and parasitic infections leading to deleterious consequences in the pathological progression of infectious diseases. Nucleotide-binding oligomerization domain (NOD)-Leucine-rich repeats (LRR)-containing receptors (NLRs), also called nucleotide-binding oligomerization (NOD)-like receptors (NLRs), are major cytosolic pattern recognition receptors (PRRs), their involvement in the orchestration of innate immunity and host defense against bacteria, viruses, fungi and parasites, often results in the cleavage of gasdermin and the release of IL-1β and IL-18, should be tightly regulated. NLRs are functionally diverse and tissue-specific PRRs expressed by both immune and non-immune cells. Beyond the inflammasome activation, NLRs are also involved in NF-κB and MAPK activation signaling, the regulation of type I IFN (IFN-I) production and the inflammatory cell death during microbial infections. Recent advancements of NLRs biology revealed its possible interplay with pyroptotic cell death and inflammatory mediators, such as caspase 1, caspase 11, IFN-I and GSDMD. This review provides the most updated information that caspase 8 skews the NLRP3 inflammasome activation in PANoptosis during pathogen infection. We also update multidimensional roles of NLRP12 in regulating innate immunity in a content-dependent manner: novel interference of NLRP12 on TLRs and NOD derived-signaling cascade, and the recently unveiled regulatory property of NLRP12 in production of type I IFN. Future prospects of exploring NLRs in controlling cell death during parasitic and microbial infection were highlighted.

## 1. Introduction

Pathogen recognition, an important initiator of host defense in the early stages of infection and subsequent generation of adaptive immune responses [1,2], is mediated by a vast repertoire of germline-encoded receptors termed pattern recognition receptors (PRR). These receptors distinguish self from non-self-molecules, i.e., pathogen-associated molecular patterns (PAMPs) [3], such as lipopolysaccharides (LPS), peptidoglycan (PGN), Lipoteichoic acid (LTA), flagellin, microbial nucleic acids, *Leishmania* Lipophosphoglycan and *P. falciparum* glycosylphosphatidylinositol (GPI) [4]. The danger/damage-associated molecular patterns (DAMPs), such as extracellular ATP, High mobility group box 1 (HMGB1), parasite hemozoin and uric acid, are recognizable by PRRs and are able to induce inflammatory signaling cascades [5]. Since the earliest report of membrane-bound Toll-like receptors (TLRs) by Jules Hoffman et al. [6] and Charles Janeway Jr [7] in *Drosophila melanogaster*, as well as the demonstration of canonical toll-like receptors in Cnidarians (Hydra) in recent decades [8], understanding of the immune system and its signal transduction have become more comprehensive. Currently, there are four major classes of PRRs: Toll-like receptors (TLRs), the Nucleotide-binding oligomerization domain (NOD)-Leucine-rich repeats (LRR)-containing receptors (NLR), the retinoic acid-inducible gene 1 (RIG-I)-like receptors (RLR; RIG-I-like helicases—RLH) and the C-type lectin receptors (CLRs). TLRs are considered to be critical in host defense, while non-TLRs are also able to recognize pathogens, to cooperate and regulate the TLRs mediated-signaling cascades that orchestrate the host immune response. Among those non-TLRs, we particularly focus on the NLR family, a series of cytoplasmic sensors with the evolutionarily conserved property, whose roles have rapidly emerged as central regulators of inflammation and immunity associated with relevant human diseases (Table 1). Therefore, the regulation of their activities has become therapeutic targets in most non-infectious and pathogenically-induced inflammatory diseases [9].

Several homologous NLR genes have been discovered in both the animal and plant kingdoms that demonstrated the plant disease-resistant genes (R gene) encoding nucleotide-binding leucine-rich repeat (NB-LRR) proteins confer the dominant resistance against diverse pathogens, thereby suggesting the conserved biological function of NLR proteins in the host defense [9]. There are approximately 22 and 34 NLR family members in humans and mice, respectively [10,11,12], and lack of specific amino acid sequence in their transmembrane domain identified them as exclusive cytosolic sensors [13]. Members of this family share common C-terminal leucine-rich repeat (LRR) domains, a central NOD (nucleotide-binding and oligomerization (NACHT) domain) for ligand recognition and a variable N-terminal effector domain, including caspase recruitment domain (CARD) or pyrin domain (PYD), or the baculoviral inhibitor of apoptosis protein repeat (BIR) domain. These receptors are chiefly expressed by immune cell lineages, such as (macrophages, neutrophils, lymphocytes and dendritic cells), as well as non-hematopoietic cells [14]. A myriad of functions have been associated with them beyond the detection of microbial components; they recognize mitochondrial DNA and ATP [15], and NLRs regulate TLRs and NOD-derived signaling cascades with the characteristic of adjuvanticity in bacteria and parasitic infections [16,17]; they are also involved in tissue homeostasis and embryonic development [18]. Similarly, NLR proteins have gained much attention in various chronic inflammatory illnesses (Table 1), autophagy [19] and carcinogenesis [20].

Recently, studies have underscored the impact of NLRs in microbial and parasitic infections. NOD1 and NOD2 play significant roles in the recognition of the bacterial peptidoglycan and provide necessary host defense against *Trypanosoma cruzi* [21] and *Plasmodium falciparum* [22]. Moreover, several species of microbes and parasites have been shown to induce the production of proinflammatory cytokines via NLRP3 and NLRC4 assemblage. The recognition of *Listeria monocytogenes* by NLRP6 for the recruitment of caspases and GSDMD cleavage has further potentiated NLRs as important molecules in cell death. Similarly, NLRP12 and NLRX1 have been demonstrated to be crucial in the regulation of interferon and cytokine productions, as well as the maintenance of immune homeostasis. The recent discovery of direct activation of noncanonical inflammasome and NLRs by cytosolic lipopolysaccharides (LPS), Lipophosphoglycan (LPG) and *Leishmania* is critical in programmed cell death (PCD). Moreover, cell death mediated by NLR inflammasome activation is executed to prevent the host cells from pathogen invasion, in which nutrient supply to pathogens is interfered [23], and the activated bystander cells could also provide the antimicrobial factors to restrict pathogen expansion and disease progression [24]. This review focuses on the significance of NLR proteins in the immune regulation after the recognition of pathogens and the programmed cell death during host defense and immune homeostasis. The potential role of type I interferon in coordinating the inflammasome activation, including the pyroptosis and the latest findings on NLRs as critical checkpoints in host innate immunity, cell death and systemic inflammation, are discussed.

**Table 1:** Involvement of the NLRs family in chronic inflammatory diseases.

Inflammatory Diseases	Affected Organs	Dysregulated NLRs Family	References
Thyroiditis	Thyroid gland	Over-expression and activation of NLRC4, NLRP1, AIM2 and NLRP3 inflammasome	[25,26,27]
Type 1 Diabetes	Pancreas	Over-expression NLRP1, NOD1/2, CIITA and NLRP3	[26,28,29]
Inflammatory bowel diseases (IBD: Ulcerative colitis and Crohn’s disease)	Gastrointestinal	NOD1, NOD2, NLRP3 and NLRP1	[26,30,31,32,33]
Celiac diseases	Small intestine	Enhanced expression of NLRP3 and CIITA, NLRP6	[26,29,34]
Autoimmune hepatitis	Liver	Hyperactivation of NLRP3 and deficiency of NLRX1	[2,26,35,36]
Arthritis	Joints	Excessive expression of NLRP3, NLRP2, CIITA, NOD2, NLRC5 and NLRP12 (beneficial), and NLRP9 and NLRP11	[25,26,37,38,39,40]
Systemic Lupus Erythematous (SLE)	Multiple organs such as Kidney, Lung and CNS	Over-expression of NOD2, NLRP3, *SNPs* in CIITA, NLRP1 and NLRX1	[26,41,42,43,44]
Vitiligo	Skin	Increased expression/activation of NLRP1 and NLRP3	[26,44,45,46]
Psoriasis	Epidermal layer (from the limbs to eyelids)	Enhanced expression of NOD2, PYCARD, CARD6, CARD14, NLRP3, NLRP1 and IFI16	[47,48,49,50]
Multiple Sclerosis	CNS: brain, spinal cord and optic nerves.	Over-activation of NOD1, NOD2 and NLRP1. Mutation in CIITA, NLRP3 and regulatory role of NLRP12, NLRC3 and NLRX1	[51,52,53,54]

## 2. Structure, Function and Classification of NOD-like Receptors

NOD-like receptors are functionally diverse intracellular sensors with heterogeneous signaling pathways. With the exception of NLRP10, which lacks an LRR domain, all the NLRs families share a common NACHT and LRR domain organization. NACHT domain possesses NTPase activity with a binding preference for GTP or ATP [55]. The structural diversity arises from variable N-terminal, which is critical for downstream signaling functions. Therefore, NLRs are phylogenetically divided into the acidic transactivation domain, pyrin domain, caspase recruitment domain (CARD) and baculoviral inhibitory repeat (BIR)-like domains, and NLRX possesses less characterized N-terminal domains usually denoted as X [56] (Table 2).

Functionally, NOD-like intracellular sensors are classified into four groups;

(i) Transcriptional trans-activators: members are CIITA and NLRC5, located at the promoter region of major histocompatibility complex (MHC) II and MHC-I, respectively [57,58]. CIITA, via its unique acidic domain (AD), is recruited to the MHC enhanceosome complex as a non-DNA binding activator to promote the transcriptional activation of MHC-II [57]. NLRC5, on the other hand, is conserved in vertebrates, with high expression in immune cells and mucosal epithelia. NLRC5 controls basal MHC I gene expression and is inducible by IFNγ stimulation to trans-activate the MHC-I gene in lymphoid and epithelial cells by reducing H3K27me3 in the MHC-I promoter [59,60,61]. The cis-regulatory elements of the promoter of the MHC-I gene interact with NLRC5 through a distinct transcriptional factor to recruit modifiers and initiate the MHC I enhanceosome transcriptional complex [60]. *Nlrc5^−/−^* mice exhibit impaired CTL responses, and NLRC5-null target cells are not efficiently cleared by CTLs, while the immunogenic melanoma was able to activate CD8+ T cells by restoring the expression of NLRC5 alongside with CD80 [56].
(ii) Activators of NF-κB and MAPK pathways: The NLRs in this category are the first described NOD-like receptors; NOD1 (NLRC1) and NOD2 (NLRC2) recognize bacterial peptidoglycans components D-glutamyl-meso-diaminopimelic acid (iE-DAP) and muramyl dipeptide (MDP), respectively, to induce the production of proinflammatory cytokines (TNF, IL-6 and IL-1β) via NF-κB and MAPK pathways [9,62]. Peptidoglycans (PGN), a constituent of the bacterial cell wall, have been demonstrated to trigger NOD2 activity sufficiently [63]. A gain-of-function mutation in the NOD2 gene is associated with autoinflammatory condition *Blau syndrome* [64] and sarcoidosis [65], whereas its loss of function is linked to Crohn’s disease [66]. Intracellular ligands recognition through the LRR domain induced the formation of a protein complex at their CARD N-terminal with an adaptor protein RIP2 for the subsequent phosphorylation of NF-κB. Moreover, the downstream signal may also go through the activation of MAPKs, including the p38, extracellular signal-regulated protein kinase (ERK) and c-Jun N-terminal kinase (JNK) pathways [10].
(iii) Inflammasome activators of the NLRs family: members of this family are involved in the inflammasome complex, i.e., an intracellular multi-protein complex that leads to the activation of caspase 1, required for the maturation of IL-1β and IL-18, as well as the amplification of NF-κB, JNK and p38 MAPK-signaling pathways [67,68]. These NLRs conduct robust secretion of proinflammatory cytokines and chemokines to the distressing site and also mediate pyroptotic cell death [57]. NLRC4 directly recruits pro-caspase 1 while intermediary cytosolic-resident adaptor apoptosis-associated speck-like protein containing a CARD (ASC) is required for PYD-carrying NLRs (such as NLRP3 and NLRP12) for the recruitment of pro-caspase 1. Aside from caspase 1, NALP1 has also been shown to participate in the activation of caspase 5 [69].
(iv) Members of the NLR family are essentially involved in the negative regulation of the proinflammatory responses by limiting IL-1β secretion, NF-κB and type I IFN (IFN-I) signaling. These include NLRP2, NLRC3, NLRP4, NLRP6, NLRP7, NLRP10, NLRP12 and NLRX1 [57]. Although the majority of NLR members here exhibit both inflammasome activation and inhibitory functions under varying conditions, ASC is recruited for the exhibition of inflammasome function; meanwhile, different endogenous proteins are engaged for the inhibitory function. Information about the NOD-like receptors in this category is scant, and their therapeutic potentials are remarkable.

Many of these NLR proteins are, individually or collaboratively, involved in detecting and triggering innate immune defenses against bacteria, viruses, fungi and parasites. Therefore, here we appraised various findings to update the current understanding of NLRs in host defense and cell death, as well as therapeutic potentials in microbial and parasitic infections.

## 3. NOD1 and NOD2 in Sensing PAMP/DAMP and Inflammatory Responses

The nucleotide-binding oligomerization domain (NOD) proteins (NOD1 and NOD2) are the first NLRs to recognize intracellular bacterial peptidoglycan directly or via immunostimulatory cargo (bacterial membrane vesicles (BMVs)) [70], *Listeria monocytogenes*, hepatitis C virus. They play important and pleiotropic roles in not only host defense against intracellular protozoan parasites, such as *Trypanosoma cruzi* [21] and *Plasmodium falciparum* [22], but also controlling the inflammation and maintenance of endoplasmic reticulum homeostasis [71]. NOD1 and NOD2 have a similar domain organization with a single N-terminal CARD domain in NOD1, and two domains are found in NOD2 (Table 2). At the steady-state, NODs exist in an auto-inhibitory state and dimerize via their NAHCT domains upon the recognition of their respective cognate ligands. This initiates the recruitment of a scaffold protein, called receptor-interacting serine/threonine kinase 2 (RIPK2), into their N-terminal CARD domain [72,73] and form a NODosome complex leading to the RIPK2 filament formation, thereby stabilizing the NODosome assemblage [74]. The NODosome complex mediates the downstream signaling activation of NF-κB, activator protein-1 (AP-1), interferon regulator factor 5 (IRF5) and the production of proinflammatory cytokines required for the pathogen clearance via ERK and MAPK pathways [75,76]. The biological functions of NOD1 or NOD2 are largely defined by posttranslational modifications, notably ubiquitination, palmitoylation and scaffolding phosphorylation [63,73,75,77]. Lu et al. found that NOD1/2 S-palmitoylation using ZDHHC5 palmitoyltransferase is critical for membrane recruitment for the effective sensing of peptidoglycan and immune signaling [78]. Several other E3 Ligases, such as XIAP, cIAP1/cIAP2, ITCH, PELLINO3, LUBAC, ZNFR4, OTULIN, TRIM27 and others [63,75,79], have been reported to regulate RIPK2 ubiquitination and NF-κB and MAPK activations during NOD1 and NOD2 signaling pathways. It is observed that NOD1 is recruited to endosomal and plasma membranes upon sensing the outer membrane vesicles (OMVs) and bacteria, respectively, whereas endosomal recruitment is maintained by NOD2 during bacterial infection.

*Nod1^−/−^* and *Nod2^−/−^* mice exhibited an increased susceptibility to several pathogens. In *Legionella pneumophila* infection, NOD1and NOD2-deficient mice showed increased infection susceptibility by dampening neutrophil infiltration in the lung and reducing the production of CXCL1/KC, IL-6 and G-CSF [80]. Similarly, NOD2 has been demonstrated to be involved in the recognition and protection against intracellular intestinal *Listeria monocytogenes* [81] and *Salmonella typhimurium* [82] by prolonging host survival with a remarkable expression of α-defensins. NOD2 also recognizes RNA and DNA viruses, such as hepatitis C virus, *norovirus*, respiratory syncytial virus, hepatitis B virus and *cytomegalovirus* [83], to trigger the phosphorylation of NF-κB and IRF3, which synergistically promote optimal IFN-β secretion [84,85]. However, Tschöpe et al. recently unveiled a contrasting role that the deficiency of NOD2 protects the *Coxsackievirus* B3(CVB3)-infected mice from the detrimental CVB3-mediated effects: the *Nod2^−/−^* mouse had reduced cardiac inflammation, less cardiac fibrosis and apoptosis compared to CVB3 infected wild-type mice [86]. This is similar to the report that NOD2 signaling contributes to intestinal inflammation and the development of colitis in patients with inflammatory bowel disease in the absence of IL-10 signaling [87]. The loss of NOD2 in IL-10-deficient macrophages reduced the production of IL-6, TNF and IL-12p40 in response to bacterial stimulation and thus dampened the usual hyper-responsiveness in the colitis mice. NOD2 participates in the coordination of both innate immune recognition and host resistance in parasitic infection. *Nod2^−/−^* mice were susceptible to parasite invasion and displayed impaired induction of proinflammatory cytokines during *Neospora caninum* infection [84]. NODs activities are also critical for IL1β, KC and IFNγ production in *Plasmodium berghei* and *falciparum* infections [71]. However, the pathogen recognition of NOD1 and NOD2 seems to be dispensable in certain conditions, as both NODs and TLRs signaling pathways lead to NF-κB and MAPK activations, and NODs impose significant impacts on innate immune signaling in the absence of TLRs. Hence enhanced NOD-signaling by priming with TLR ligands, such as LPS or viral infection, is also achievable in an IFN-I dependent manner in vitro and in vivo [63,88]. Together, these reports suggest the importance of NODs in pathogen recognition, host defense and collaboration with TLR signaling in driving innate immunity [89,90].

The link between NOD1 and ISGF3 signaling pathways is another remarkable finding reported in *Helicobacter pylori*-infected human epithelial cells. This study described that the binding of a ligand with NOD1 triggered the activation of serine-threonine kinase RICK, which then bound to TNF receptor-associated factor 3 (TRAF3), in turn leading to the activation of TANK-binding kinase 1 (TBK1) and IκB kinase ε (IKKε) for the subsequent activation of interferon regulatory factor 7 (IRF7) [91]. IFN-β production driven by IRF7 contributes to the activation of a heterotrimeric transcription factor complex, known as interferon-stimulated gene factor 3 (ISGF3), consisting of phosphorylated STAT1, STAT2 and IRF9, which subsequently drives the production of CXCL10 and additional IFN-I [92]. After a decade, this pathway was confirmed in the virus with the addition that NOD1 acts as a positive regulator of the MDA5/MAVS complex downstream of the TRAF3 pathway during spring viremia of carp virus (SVCV) infection [93]. A connection between NOD signaling and cell death appears to be possible because their N-terminal CARD domain may recruit caspases, and many protein complexes involved in the NOD signaling pathway are closely associated with cell death [79,90], but no study has ever established a direct link between NODs and Gasdermin D (GSDMD)-mediated pyroptosis [79], except autophagy. It was shown in an overexpression system that NOD2 could bind to multiple caspases via its CARD and was able to directly activate caspase-9 and induce apoptosis [94]. A report also showed that MDP stimulation promotes NOD2 [95] and/or in complex with NALP1 [96] to induce caspase-1-dependent IL-1β secretion and autocrine signaling [95,96]. These reports suggest that the dominant role of NODs is in immunomodulatory and host defense rather than cell death.

## 4. NLRP3 and NLRC4 Inflammasomes

The NLR family pyrin domain-containing protein 3 (NLRP3), a tripartite protein (as described in Table 1), and extensively reviewed in the sub-family of inflammasomes [97,98]. Cellular events, such as ionic flux, the release of ROS, mitochondrial dysfunction and lysosomal damage, have been reported to induce NLRP3 inflammasome activation. Its activation mediates caspase 1-dependent IL-1β and/or IL-18 secretion, and GSDMD dependent pyroptosis. NLRP3 inflammasome comprises NLRP3, adaptor molecule apoptosis-associated speck-like protein containing CARD (ASC, also known as PYCARD) and caspase 1. The oligomerization of NLRP3 at NACHT domains occurs upon stimulation, followed by the recruitment of ASC through hemolytic PYD-PYD. Multiple ASCs merge to further recruit caspase 1 via CARD–CARD interactions to enable self-cleavage and the activation of caspase 1 [97]. The activation of NLRP3 can be through canonical and noncanonical pathways [99]. Two steps are required in canonical activation: priming/transcription and activation/assemblage, whereas priming is not necessary for noncanonical activation. Priming in the canonical pathway goes through toll-like receptors (TLRs), tumor necrotic receptors (TNFR) [100], C-lectin receptors (CLRs) [101] and IFN receptors (IFNAR) [102,103] for the activation of the nuclear factor kappa B (NF-κB) pathway; thus, leading to the transcriptional upregulation of NLRP3 (ready for and post-transcriptional modifications (PTMs), such as ubiquitination and phosphorylation) and pro-IL-1β proteins in the cytosol for ASC recruitment [100]. The activation signal for conformation change comes from pathogen and sterile activators (otherwise called PAMPs and DAMPs), examples include: nigericin, extracellular ATP, silica, cholesterol crystals, potassium efflux and reactive oxygen species (ROS). Thereafter, mature caspase 1 will simultaneously induce the cleavage of GSDMD to promote the pyroptotic cell death and the release of mature cytokine proteins IL-1β and IL-18. In contrast, noncanonical activation of NLRP3 bypasses the transcriptional priming and based on direct activation of the noncanonical inflammasome (caspase 4/5/11) in response to the endotoxin LPS from Gram-negative bacteria [104], *Leishmania* Lipophosphoglycan (LPG) [105] and oxidized phospholipid1-palmitoyl-2-arachidonoyl-sn-glycero-3-phosphorylcholine(oxPAPC) [106,107]. Noncanonical inflammasome induces cell death and cleavage of autoinhibited GSDMD into C- and N-terminals; the released N-terminals oligomerize and assemble at the plasma membrane to cause membrane damage and also facilitate potassium efflux and pyroptosis, as well as enhanced NLRP3 inflammasome activation [104,108]. Therefore, the intrinsic execution of pyroptosis by noncanonical inflammasome requires NLRP3, ASC and caspase 1 for the maturation and secretion of cytokines. Caspase 1 and caspase 4/11 can induce pyroptosis mediated-IL-1β secretion, but caspase 4/11 cannot directly cleave pro-IL-1β and pro-IL-18, but enhance caspase 1 activation for this function instead [109]. Recently, there are new reports demonstrating a novel role of caspase 8 and FADD in the regulation of NLRP3 inflammasome activation and maturation of IL-1β [110,111,112]. Kang et al. demonstrate that caspase 8-deficient dendritic cells (DC) exhibit enhanced LPS-induced NLRP3 assembly [113], and also similar finding occurred in macrophage during *Candida albicans* infection [114]. However, non-apoptotic caspase 8 seems to play an essential role in TLR signaling-mediated *Nlrp3* priming [115]. Another study further showed that FADD-caspase 8 is a critical upstream regulator in both canonical and noncanonical NLRP3 inflammasome signaling, as well as in transcriptional priming [116]. In macrophages, the deletion of caspase 8 in the presence or absence of RIPK3 inhibited the caspase 1 and caspase 11 activation by NLRP3 stimuli. FADD is positioned upstream of caspase 8, and whose deletion prevents caspase 8 maturation. Mice deficient in FADD and caspase 8 exhibited impaired IL-1β production during *C. rodentium* infection or while being challenged by LPS [116].

NLRP3 inflammasome activation has been overwhelmingly reported to mediate innate immune response in many intracellular pathogens. In gram-positive bacterial infections, NLRP3-dependent caspase 1 regulates the acidification of phagosome buffering by NADPH oxidase NOX2 to modulate innate immune response [117]. Hyperactivation of NLRP3 via gain-of-function mutation in the *Nlrp3* gene (Nlrp3R258W) promotes the clearance of virus H1N1 IAV infection. This efficacy is ascribed to IL-1β dependent neutrophil recruitment [118], but this response was deleterious in the H7N9 viral challenge [119]. Pyroptosis mediated by NLRP3 inflammasome plays an essential role in HIV-1–infected patients whose CD4+ T cells are lost [120]. This is similar in parasitic infections, such as *Trichuriasis* [121], *Neosporosis* [122], *Leishmania* [123,124], Chagas disease [125,126], *Toxoplasmosis* [127], *Trichomoniasis* [128] and invasive *Entamoeba histolytica* [129], as well as in fungal infections [130,131,132]. A variety of the corresponding cognate ligands and post-transcriptional modifications exhibited by NLRP3 have been extensively reviewed elsewhere [97,98]. In parasitic infection, the recognition of *Trichuris* antigen and exosome by NLRP3 conciliates the secretion of IL-18 that promotes parasite persistence. *Nlrp3*^−/−^ mice with reduced proinflammatory type 1 cytokine responses and augmented protective type 2 immunity results in worm expulsion [121]. Extracellular *Entamoeba histolytica* also induces NLRP3-dependent caspase 1 via its contact with α_5_β_1_ integrin at the Macrophage-*Amebae* intercellular junction. The α_5_β_1_ integrin induced ATP release into the extracellular space through the opening of pannexin-1 channels that signaled through P2X_7_ receptors to deliver a critical co-stimulatory signal that activates the NLRP3 inflammasome for the induction of IL-1β [129]. Likewise, ROS production was induced after *Neospora caninum* to mediate NLRP3 activation and the production of proinflammatory cytokine in macrophages [122].

**Figure 1:**
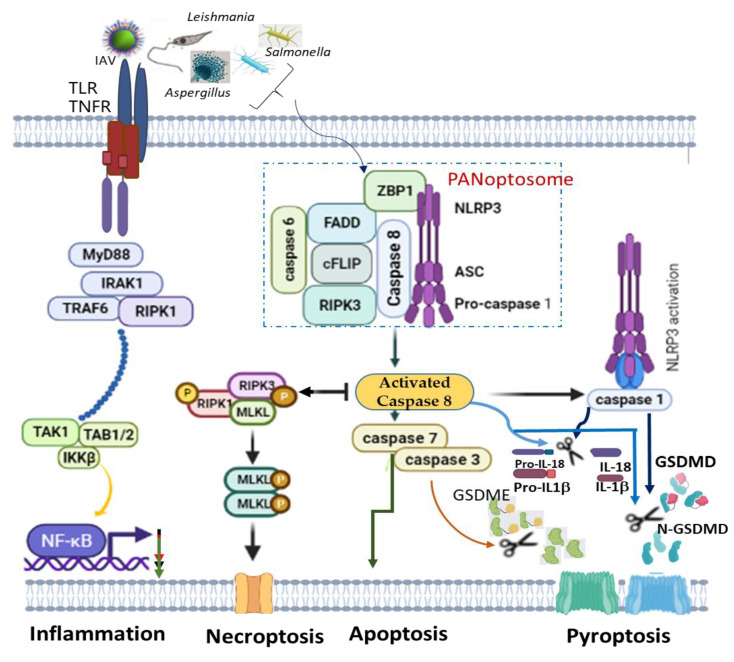
NLRP3 is an essential component of PANoptosome, and activation of a complex of ZBP1-FADD-RIPK3-caspase 8 drives PANoptosis inflammatory cell death. Activated caspase 8 simultaneously induces apoptosis via downstream activation of caspase 3/7 and directly promotes the assemblage of NLRP3 inflammasome activation complex to initiate pyroptosis via the cleavage of pro-IL1β, pro-IL18 and GSDMD. The reduced activity of the activated caspase 8 drives phosphorylation of MLKL for the induction of necroptosis, while pathogen surface recognition through TLR and TNFR leads to the nuclear binding of NF-κB for inflammation and PANoptosome-independent necroptosis. NLRP3, Nucleotide-binding oligomerization domain; GSDMD, Gasdermin D; GSDME, Gasdermin E; ZBP1, Z-DNA-binding protein 1; RIPK, Receptor-interacting serine/threonine kinase; MLKL, mixed lineage kinase domain-like pseudokinase; FADD, fas-associated death domain; MyD88, myeloid differentiation primary response 88; NF-κB, nuclear factor kappa light chain enhancer of activated B cells; IRAK, interleukin receptor-associated kinase; TAK1, transforming growth factor b-activated kinase 1; TRAF, TNF receptor-associated factor; cFLIP, Cellular caspase-8 (FLICE)-like inhibitory protein.

A recent finding on PANoptosis (concomitant activation of three autonomous cell deaths: pyroptosis, apoptosis and necroptosis) also identified NLRP3 as one of the indispensable components of PANoptosome (Figure 1). PANoptosis is a programmed cell death (PCD) common in macrophages infected with intracellular pathogens, such as influenza A *virus, vesicular stomatitis* virus, *Listeria monocytogenes*, *Salmonella enteric serovar Typhimurium*, *Candida albicans* and *Aspergillus fumigatus*, to circumvent pathogen-mediated inhibition [133,134]. The recognition of pathogens or their products by ZBP1 mediates the assemblage of a cytoplasmic multimeric protein complex, known as PANoptosome, and induces PCD called PANoptosis. This complex comprises NLRP3, ASC, CASP8, RIPK3, CASP6 and Z-DNA binding protein 1 (ZBP1) [134,135,136]. ZBP1, which is a DNA-dependent activator of IFN-regulatory factors (DAI) [137], interacts with NLRP3 inflammasome, and together with CASP8, to form a PANoptosome [138,139]. Simultaneous deletion of NLRP3 inflammasome components and caspase 8 largely rescued multiple PCDs than an individual deletion, which provided reduced or no redemption of PCD in pathogen-infected macrophages [140]. This is simply because caspase 8 can exhibit non-apoptotic function to directly cleave pro-IL-1β, pro-IL-18 and GSDMD as well as promotes GSDME-mediated pyroptosis via downstream caspase 3 and caspase 7 activations [136]. However, even though caspase 8 is an essential modulator of PANoptosis, its shifting towards necroptosis and pyroptosis is possible alongside RIPK3 and NLRP3 activation, respectively [141,142] (Figure 1).

NLRC4, NLR family CARD domain containing 4 formerly called IPAF (ICE protease-activating factor) for its ability to activate caspase 1, is another canonical inflammasome that is tightly regulated by transcriptional and posttranscriptional mechanisms. Its expression is upregulated by TNF and the stress-mediated p53 activation [143,144,145]. NLRC4 contains a CARD domain, and therefore, can directly recruit pro-caspase 1 via CARD–CARD interaction to activate caspase 1, associates or co-localizes with ASC [143,146] for proteolytic cleavage of proinflammatory cytokines (pro–IL-1β and pro–IL-18) and GSDMD [147,148]. The pathogen recognition of NLRC4 is usually via a sensor NAIP (NLR family, apoptosis inhibitory proteins) [149]. Mouse NAIP5/NAIP6, NAIP2 and NAIP1 specifically recognize bacterial flagellins, TTSS rod and T3SS needle proteins, respectively, to induce the activation of NLRC4 inflammasome [149,150,151]. Human NAIP merely recognizes T3SS rod protein PrgJ [152], and its splice variant senses flagellin to promote NLRC4 inflammasome activation in humans [153]. The NLRC4-dependent caspase 1 activation and cell death are cell-specific. NLRC4 activation is critical in macrophages infected by intracellular invasive bacteria *Salmonella typhimurium* and *Burkholderia thailandensis*, which mediates pyroptotic cell death and the release of proinflammatory cytokines: IL-1β and IL-18 [154,155,156]. However, unlike macrophages, NLRC4 activation in neutrophils was found to be critical for caspase 1-dependent IL-1β production but not pyroptotic cell death [104,154]. Pyroptotic cell death in neutrophils is majorly coordinated by noncanonical inflammasomes (caspase 4/5/11). A study also revealed that NLRC4 mediates IL-18 secretion necessary to drive IFN-γ, which subsequently primes both macrophages and neutrophils for caspase 11 activation during *Burkholderia thailandensis* infection in mice [155]. In addition, leucine-rich repeat kinase 2 LRRK2 is an intrinsic regulator of NLRC4 and LRRK2 formed a complex with NLRC4 for its optimal phosphorylation at Ser533, which promotes inflammasome activation during *S. typhimurium* infection in macrophages [157]. Interestingly, just as a report showed, acetylation-induced NLRP3 activation can be reversed by SIRT2 [158]; also SIRT3 was found to promote NLRC4 inflammasome activation by deacetylation [148]. The involvement of NLRC4 in pathogen recognition was largely thought to be restricted to bacterial infections but recent studies revealed that dendritic cell NLRC4 regulates T cell response during influenza A virus infection. Similarly, NLRC4 was demonstrated to promote the susceptibility of *Paracoccidioides brasiliensis* (pathogenic fungus infection) by regulating NLRP3 activities to dampen the late IL-18 production and CD8+ IFN-γ+ T cell responses [159].

## 5. NLRP6 and NLRP10 in Regulatory and Inflammatory Responses

NLRP6: It is originally called PYRIN-containing Apaf-1-like proteins 5 (PYPAF5), a cell-specific function receptor that coordinates a synergistic activation of NF-κB and the activated caspase 1-dependent cytokine processing when co-expressed with ASC [160]. It is chiefly expressed in epithelial cells, fibroblasts, granulocytes, dendritic cells, macrophages and CD4+ and CD8+ T cells [161]. Human and mouse epithelial cells treated with TNF and rosiglitazone, an agonist of PPARγ (transcriptional factors peroxisome proliferator-activated receptor-γ), exhibited enhanced NLRP6 expression. NLRP6 is transcriptionally regulated by a series of stimuli, including synthetic polyinosinic:polycytidylic acid (poly(I:C)), encephalomyocarditis virus, LPS, MDP and iE-DAP, as well as IFN-α in many types of cells [162,163]. As an NLR inflammasome, NLRP6 has been shown to form multi-protein complexes with ASC for the maturation and production of IL-1β and IL-18 [161,164], as evident in a dextran sulfate sodium (DSS)-induced colitis model [164,165]. Excessive inflammation by NLRP6-dependent IL-18 production could be regulated by CYLD, which mechanistically deconjugates the K63-linked ubiquitin chains on NLRP6 to prevent an NLRP6–ASC inflammasome complex [166]. The impact of NLRP6 inflammasome activation is cell- and pathogen-dependent. In pulmonary *S. aureus* infection, deleterious pyroptotic and necroptotic cell deaths exhibited by wild-type macrophages were attributed to the uncontrolled NLRP6 inflammasome activation that mediates caspase 1 activation and IL-1β production [167]. In contrast, NLRP6 inflammasome activation mediated IL-1β production is required for host survival and bacterial clearance, as well as neutrophil in-flux in a *Klebsiella pneumoniae*-induced pneumonia-derived sepsis [18].

Intriguingly, Hara et al. recently reported that *Listeria* and Lipoteichoic acid (LTA) sensed by NLRP6 promotes the IFN-I-dependent noncanonical inflammasome (caspase 11), as well as caspase 1 activation, via ASC adaptor protein [168]. The induction of NLRP6-ASC-caspase 11 complex by LTA can further promote caspase 1 activation and inflammatory cytokines’ (IL-1β and IL-18) maturation in macrophages. The reconstitution of IL-18 caused an outrageous outcome in *Nlrp6^−/−^* and *Casp 11^−/−^* mice by *Listeria* infection, suggesting that the involvement of IFN-I and caspase 11 in the production of IL-18 may worsen *Listeria* infection outcome. Similar reports revealed that the recruitment of caspase 1 and caspase 11 by NRLP6 activation occurred in the *Streptococcus pneumoniae* infected macrophages [169], with the addition that the activation of NLRP6 reduced the activation of NF-κB and ERK signaling pathways. In parasitic infection, severe inflammation ascribed to soluble Egg Antigen (SEA) of *Schistosoma mansoni* (causative agent of gastrointestinal schistosomiasis) is crucially mediated by NLRP6 inflammasome activation, which intensifies chemokines production (CXCL1/KC, CCL2, CCL3, IL-5 and IL-10) and immune cell recruitment into the liver. Caspase 1 and IL-1β in the liver dendritic cells are critical for GSDMD-dependent hepatic granuloma, resulting in periovular inflammation and collagen deposition [170]. In contrast, the release of IL-18 via enterocyte NLRP6 inflammasome activation is necessary for parasite clearance in intestinal infection caused by *Cryptosporidium tyzzeri* [171]. The detection of *Cryptosporidium* sporozoite and trophozoite induces ASC-dependent NLRP6 inflammasome activation, and GSDMD-dependent IL-18 is deficient in NLRP6-deficient mice, leading to the increased susceptibility to *Cryptosporidium* infection [171].

The clarification on possible redundancy of NLPR6 in the presence of an NLRP3 inflammasome study revealed that inflammasome assemblage of NLRP6 is independent of NLRP3 with the observation that NLRP3 activation was intact in *Nlrp6^−/−^* bone marrow-derived macrophages (BMDMs) and enhanced IL-1β production is obtainable in these macrophages when challenged with NLRP3 agonists (ATP and Nigericin) [167]. Moreover, unlike NLRP3, which activates caspase 1 alone, NLRP6-dependent pyroptosis is also regulated by IFN-I. In addition, NLRP6 also senses viral RNA (*encephalomyocarditis virus* and murine norovirus), leading to ATP-dependent interactions between RNA helicase DEAH (Asp-Glu-Ala-His) box helicase 15 (Dhx15) and mitochondrial antiviral signaling protein for the induction type I/III interferons (IFNs) and IFN-stimulated genes (ISGs), such as the transcription factors IRF3 and IRF7 [162,163]. Similarly, some reports have also described NLRP6 as a negative regulator of innate immunity through NF-κB and MAPK signaling pathways to limit the inflammation and pathological damage upon the recognition of pathogen and pathogen products in most of the myeloid cells, such as neutrophil, macrophage and inflammatory monocyte [172,173]. Anand et al. [173] demonstrated that NLRP6 specifically inhibits TLR2- and TLR4-downstream signaling cascade to limit excessive production of TNF and IL-6 inflammatory cytokines. A report further showed that inflammasome activation of NLRP6 results in the reduced NF-κB and ERK signaling pathways [169]. Therefore, this suggests that inflammasome activation and immunity regulatory functions may occur concurrently.

NLRP10: It is one of the less-studied NOD-like receptors and is widely expressed in myeloid cells, epithelial cells and keratinocytes [174]. The unique feature of NLRP10 is the lack of a leucine-rich repeat domain to participate in ligand sensing or binding; thus, it is speculatively thought to be involved in the regulation of other NLRs. The earliest report showed that it interferes with the ASC domain of NLRP3 to inhibit caspase 1 and IL1β-mediating cell death and also interacts with NOD1 pathways (such as RIP2, TAK1 and NEMO) to regulate the induction of proinflammatory responses via NF-κB activation during *S. flexneri* infection [175,176]. However, recent reports have challenged these speculations in NLRP3 inflammasome: it was found that IL1β, IL-6 and TNF were not affected in macrophages from *Nlrp10^−/−^* mice treated with NLRP3 agonist but significant impairment in the adaptive immunity was observed in *Nlrp10^−/−^* mice instead [177]. This result suggests that NLRP10 does not function to regulate NLRP3 inflammasome. It would rather be critical in T-helper cells and systemic antibody production because a loss of NLRP10-impaired DC migration to the lymph node due to intrinsic defect [177] also correlates with *Candida*-specific Th1 and Th17 defects [178]. Until now, the role of NLRP10 in host immune responses during microbial infection and cell death is controversial and thus requires further investigations. A study has described its involvement in the inflammatory resolution during cutaneous *Leishmania major* infection by the regulation of neutrophil homeostasis [174].

## 6. NOD-Like Receptors in the Regulation of IFN-I and Proinflammatory Responses

NLRX1: is a non-inflammasome and negative regulator of NF-κB and IFN-I. It regulates the reactive oxygen species (ROS) production, as well as amplifying NF-κB and JNK activations [2,179]. This mitochondrially-located intracellular receptor lacks a well-characterized N-terminus and serves as the central gatekeeper between mitochondrial biology and immunological response. NLRX1 has been implicated in many infectious and sterile inflammatory diseases; however, its connection to autoimmunity and cancer biology is raising NLRX1 as a strong therapeutic target [41]. NLRX1 is structurally complex, a less characterized N-terminus (often denoted as “X”) and seven LRRs followed by an uncharacterized three-helix bundle. The enigmatic structure termed X in the N-terminus contains only one identified domain, called the mitochondrial targeting sequence (MTS), which attaches NLRX1 to the mitochondrial membrane [180]. Similar to other NLRs, NLRX1 can shuttle within the cellular compartments, from the cytosol to the mitochondria to initiate and complete its signal pathways [1,2]. The subcellular relocalization is prompted by infections; NLRX1 is reported to be translocated from the cytoplasm to the mitochondria of epithelial cells during *Rhinovirus* infection [181] and phagosome in fungal (*Histoplasma capsulatum*) infection [182].

The interaction of NLRX1 with other proteins informs a specific function in different cell compartments: it interacts with UQCRC2 for ROS production in the mitochondrial matrix [178] and FASTKD5 for the maturation of mitochondrial precursor transcripts [183]. It sequesters the DNA-sensing adaptor STING from TANK-binding kinase 1 (TBK1) in the endoplasmic reticulum to modulate an innate immune response to HIV-1 and DNA viruses [184] and interferes with TRAF6 and IKK complexes in the cytoplasm for the attenuation of NF-κB [185,186]. Essentially, NLRX1 halts the interaction between dsRNA-activated RIG-I and MAVS to negatively regulate mitochondrial antiviral signaling protein (MAVS)-mediated type I IFN signaling via TRAF3 and TRAF6 ligase ubiquitination [2,186,187]. This is evidently observed in *Nlrx1^−/−^* mice that exhibited increased expression of IFN-β, STAT2, OAS1 and IL-6 after influenza virus infection [187]. Similar studies also reported that NLRX1 interacts with mitochondrial Tu translation elongation factor (TUFM) to attenuate IFN-I and enhance autophagy in *Vesicular stomatitis virus* (VSV) infection [188].

**Figure 2:**
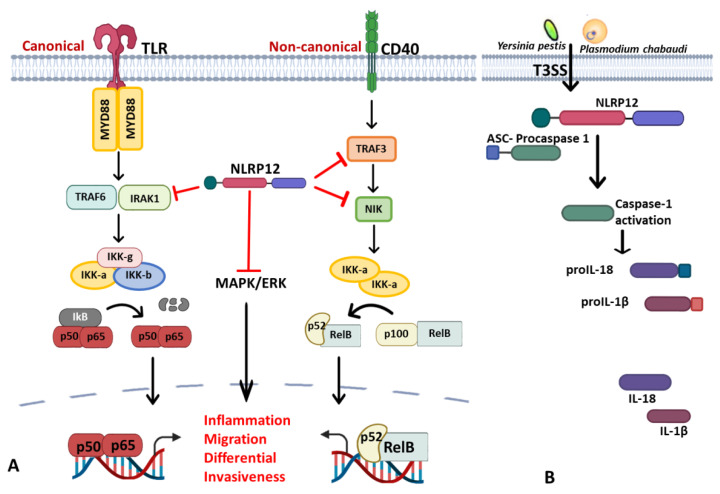

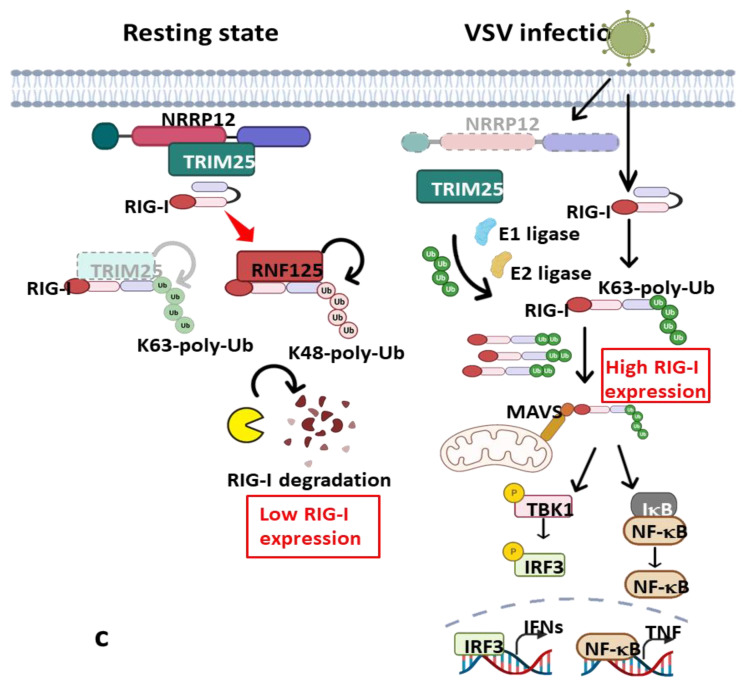
NLRP12 negatively regulates innate immune signaling in a pathogen-dependent manner. (**A**) NLRP12 inhibits IRAK1 and TRAF3/NIK to further inhibit canonical and noncanonical NF-κB signaling and the MAPK/ERK inflammatory signaling pathway in bone marrow-derived macrophages (BMDMs). (**B**) NLRP12 participates in the recruitment of ASC for the processing of caspase 1 and maturation of IL-1β and IL-18 to positively regulate innate host defense during Yersinia pestis and Plasmodium chabaudi. (**C**) NLRP12 associates with TRIM25 to reduce polyubiquitination of RIG-I and inhibits the RIG-I-mediated IFN response during VSV infection in bone marrow-derived dendritic cells. RIG-, Retinoic acid-inducible gene I; MAVS, Mitochondrial antiviral-signaling protein; TBK1, TANK-binding kinase 1.

NLRP12: (also known as CLR19.3, Monarch1, NALP12, PAN6, PYPAF7 and RNO2) is another pyrin domain-containing NLR receptor that is predominantly expressed in cells of the myeloid lineage, chiefly neutrophils, dendritic cells and monocytes. Similar to NLRP6, it has inflammasome and non-inflammasome dependent functions, though its specific stimuli are still unknown, its expression is often down-regulated by pathogens, pathogen products and inflammatory cytokines [57,189,190], especially the agonists of Toll-like receptors (TLRs) [191]. Mutation of the NLRP12 gene has been associated with NLRP12AD (autosomic dominant disease), characterized by recurring cold fever with headache, lymphadenopathies, oral ulcers and abdominal pain [192]. Studies so far have categorized the functions of NLRP12 under the following headings:

(i) Regulator of canonical and noncanonical NF-κB and MAPK signaling pathways: NLRP12 is a negative regulator protein that inhibits canonical and noncanonical activations of NF-κB and ERK (Figure 2A). *Nlrp12^–/–^* mice show exaggerated NF-κB activation and ERK phosphorylation in colitis-associated colorectal cancer models [193], osteoclast differentiation [194] and in bone marrow-derived macrophages treated with *Mycobacterium tuberculosis* [190] and *Salmonella* LPS but not flagellin [195]. Canonical interference of NLRP12, as demonstrated by Zaki et al., is via the suppression of hyperphosphorylation of IRAK1 to limit of IκBα and ERK phosphorylation downstream of TLR-MyD88, thus reducing nuclear translocation of NF-κB and secretion of proinflammatory cytokines in macrophage stimulated with *S. typhimurium* [195]. Similarly, its interaction with NF-κB-inducing kinase (NIK) and TRAF3 was through its NOD and LRR domains, leading to proteasomal degradation of NIK in noncanonical NF-κB signaling [33,57,190] in microbial and parasitic (*Leishmania major*) infections [196]. Therefore, the constitutive elevation of NIK, processing of p100 to p52 and reduced degradation of TRAF3, was observed in *Nlrp12^–/–^* cells [33]. These studies recapitulate NLRP12 as a potential checkpoint for NF-κB signaling in murine macrophages and human THP-1 monocytic cells by negatively regulating both TLR and TNFR pathways [33]. In fact, NLRP12 also exhibits its inhibitory role by degrading NOD2 through the ubiquitin–proteasome pathway to raise host tolerance towards bacterial muramyl dipeptide (MDP) by sequestering heat-shock protein 90 (HSP90). Sequestration of HSP90 prevents the stabilization of NOD2/RIPK2 complex in response to MDP, thus repressing NOD2 signal transduction of NF-κB and subsequent activity of the JAK/STAT signaling pathway [197]. The physiological impact of NLRP12 regulation in the immune response is still elusive, for instance, the loss of NLRP12 in BMDC induces IL-6 and TNF upon *M. tuberculosis* or *Klebsiella pneumonia* without conferring resistance against these bacteria [198]. In hepatocellular carcinoma (HCC), however, NLRP12 downregulates the JNK-dependent inflammation and proliferation of hepatocytes and NLRP12 deficient mice were highly susceptible to diethyl nitrosamine (DEN)-induced HCC with increased inflammation, hepatocyte proliferation and tumor burden. In contrast, the upregulation of NLRP12 was reported in response to *Porphyromonas gingivalis* LPS in RAW264.7, and its depletion in the cell line corresponds to an increase in TNF production and iNOS expression [199].
(ii) A negative regulator of Type-I interferon and proinflammatory responses: This is another remarkable function of NLRP12 that was recently demonstrated by our research group; we reported an interference of NLRP12 on RIG-I-mediated IFN-I production during *vesicular stomatitis virus* (VSV) [200]. We found that VSV infection downregulates NLRP12 expression, and its deletion in BMDC provokes severe transcription and production of IFN-I (IFNα/β) and TNF that corresponds with reduced viral titer and relative genomic copy in *Nlrp12^–/–^* DCs upon infection. In the infected DCs, TRIM25, an E3 ligase required for Lys63-linked polyubiquitination and activation of RIG-I, mediates the downstream activation of MAVS (Figure 2C). MAVS associates with the adaptor protein TRAF3 and TRAF family member-associated NF-κB activator (TANK) to trigger the activation of TANK-binding kinase 1 (TBK1) and IκB kinase, leading to the activation of IRFs and production of IFN-I and TNF; thus, enhanced immune signaling cascades were observed in *Nlrp12^–/–^* DCs treated with VSV and 5′ppp dsRNA. However, the presence of NLRP12 relieved the binding of TRIM25 with RIG-I to suppress IFN-I production. Mechanistically, NLRP12 promotes RNF125-mediated degradation of RIG-I by associating with ubiquitin ligase TRIM25 to reduce K63-linked ubiquitination of the antiviral innate immune receptor RIG-I (Figure 2C). This will ultimately prevent RIG-I association with MAVS to checkmate the transcription and secretion of interferon and cytokine induction in response to RNA viruses. Domain mapping analysis showed that the NBD domain is presumably a critical target for TRIM25 interaction [200]. *Nlrp12^–/–^* mice are more resistant to VSV infection with lower viral loads in the brain and recover faster than WT mice with less neuronal loss in the ventral striatum and hypothalamus in *in vivo* study.
(iii) NLRP12 inflammasome and its positive regulatory property in other inflammasomes: The foremost inflammasome functions of NLRP12 were reported in an overexpression system where NLRP12 co-expressed with ASC for caspase 1 and IL-1β production [201]. In primary cell and animal study, NLRP12 is involved in the caspase 1-mediating production of inflammatory cytokines (IL-18) and is crucial for the host defense against *Yersinia pestis* infection; thereby, deficiency of NLRP12 causes the susceptibility to *Yersinia pestis* infection as it occurred in the IL-18 deficient mice [202]. The actual ligand sensed by NLRP12 in *Yersinia* for its activation is not known, but it was noted that ligand generation requires a complex type III secretion system (T3SS) (Figure 2B).The inflammasome assemblage of NLRP12 was demonstrated in the dendritic cells from spleen and bone marrow treated with *Plasmodium chabaudi*, where NLRP12 was collaboratively required for ASC-dependent caspase 1 for the systemic production of IL-1β and pyroptosis [203]. Similarly, collaboration of NLRP12 with other inflammasomes was reported in pyroptosis mediating ganglion cell death of acute glaucoma, NLRP12 collaborates with NLRP3 and NLRC4 to elicit pyroptotic processes and IL-1β maturation through caspase 1 activation [204]. Not only that, simultaneous expression of the NLRP3, NLRP12 and IFI16 inflammasomes in cornea infection induced by virulent HSV-1 strains is ascribed to the enhanced caspase 1, IL-1β and IL18 alongside with co-expression of dense specks of the adapter molecule ASC [205]. However, a contrary report was obtained during *Brucella abortus* infection that portends NLRP12 as an anti-inflammatory regulator that inhibits not only NF-κB and MAPK signaling but also caspase 1 activation in BMDMs, and its absence conferred the host resistance in murine brucellosis [206]. All these indicate that the function of NLRP12 is stimuli-dependent, and its collaboration with other NLRs may be partially ascribed to dearth of specific ligands to be sensed. However, evidence-based reports have described it as a critical checkpoint in innate immunity in microbial and parasitic infections by regulating innate immune signaling cascades negatively or positively. Since its function varies with pathogens, it is pertinent to investigate the role of NLRP12 in other pathogens.

## 7. NOD-Like Receptors in the Regulation of Pyroptosis Cell Death

Pyroptosis is a lytic programmed cell death (PCD) that involves cell swelling and the rupturing of the plasma membrane, and it remains a major pathway for the release of proinflammatory proteins in macrophages [207], dendritic cells and partially in neutrophils [208,209]. This cell death was first observed by Friedlander et al. in 1986 in primary mouse macrophages with anthrax lethal toxin (LT) treatment leading to the rapid release of cell contents [210] and later established in *Shigella flexneri*-infected macrophages by Zychlinsky and his co-workers in 1922 [211].

The establishment occurred after the discovery of ICE (interleukin-1β-converting enzyme) otherwise called caspase 1 [212] as a critical weapon for the maturation of IL-1β from the precursor [213,214]. However, the phenomenon was first thought to be apoptosis until 2001 when D’Souza et al. coined the term pyroptosis as proinflammatory programmed cell death to distinguish it from non-inflammatory cell death called apoptosis [215,216].

**Figure 3:**
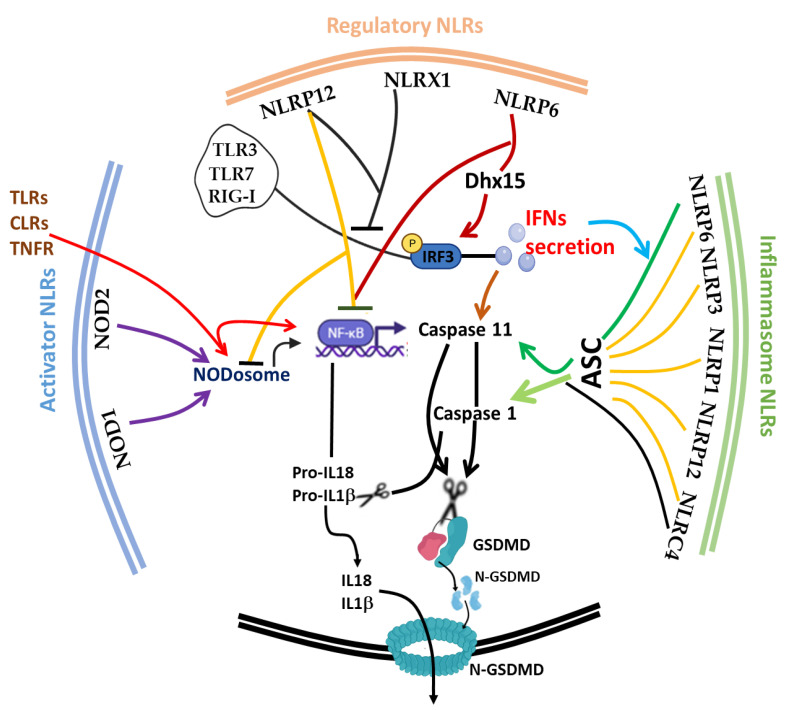
The interplay between the principal molecules of pyroptosis and NOD-like receptors: Caspase 1, GSDMD, IL-1β, IL-18, caspase 11 and IFN-I are principal mediators of pyroptosis. Caspase 1 and 11 cleave GSDMD and the N-terminal GSDMD loads onto the plasma membrane to form a pore and promotes the release of IL-1β and IL-18. The inflammasome NLRs are the upstream sensors for caspase 1 activation via their complex formation with ASC and transcriptional basal level of ASC, inflammasomes, such as NLRP3, caspase 11 and immature cytokines (pro-IL1β and pro-IL18), are upregulated along with NF-κB induced downstream of TLRs, CLRs, TNFR and NODosome signaling pathways. Cytosolic recognition of pathogens by interferon-related receptors, such as TLR3, TLR7 and RIG-I, also induce IFN-I production, which can prime NLRP6 for caspase 11 activation, and then caspase 11 further enhances the caspase 1-mediated pyroptosis. Members of regulatory NLRs act to suppress the pathway of each critical molecule; for instance, NLRP12 and NLRX1 interfere with RIG-I signaling cascade, canonical and noncanonical NF-κB pathways. TLRs, Toll-like receptors; CLRs, C-type lectin receptors; TNFR, Tumor necrosis factor receptor 1; RIG-I, Retinoic acid-inducible gene I; TLR3/7, Toll-like receptor 3/7; IFNs, Type 1 interferon; NOD1/2, Nucleotide-binding oligomerization domain-containing protein 1/2; NLRP1/3/6/12, NOD-like receptor family pyrin domain-containing 1/3/6/12; NLRX1, Nucleotide-binding oligomerization domain, leucine-rich repeat containing X1; IRF3, Interferon regulatory factor 3; ASC, Adapter apoptosis-associated speck-like; GSDMD, Uncleaved Gasdermin-D; N-GSDMD, Cleaved N-terminal Gasdermin-D.

The discovery of inflammasome in 2002 [217] made it clearer that efficient execution of pyroptosis requires the activations of the intracellular inflammasome signaling complex to activate inflammatory caspase 1 necessary for the process of IL-1β and IL-18 from pro-IL-1β and pro-IL-18, respectively. Until 2015, when gasdermin D was discovered as a substrate target of caspase 1/4/5/11, the pyroptosis effector molecule was unknown [218,219]. The oligomerization of the cleaved active form of gasdermin protein (N-terminal of GSDMD) [217,218] results in the lysis of cells and facilitates the release of proinflammatory cytokines (such as IL-1β, IL-18 and TNF) and alarmins, such as high mobility group box 1 (HMGB1) [207,208]. It is noteworthy to mention that the first report of the gasdermin gene (now GSDMA) in the gastrointestinal tract (tightly restricted to the esophagus and stomach) and skin of a mouse was 2000 [220], and since then, many members of the gasdermin family have been reported; GSDMB, GSDMC, GSDMD, GSDME (also known as DFNA5) and PJVK (also known as DFNB59), with different activating enzymes both in human and mouse [221,222,223]. This expansive advancement in the understanding of the gasdermin family and its activating protease enzymes, such as inflammatory caspase 4/5/11 [112,221], non-inflammatory caspase 3/7/8 [222,223], Cathepsin G [224] and neutrophil elastase [225], as well as inflammasome activation [226,227], has further broadened the concept of cell death as critical therapeutic targets [228,229] in host immunity [230,231], microbial-induced hyperinflammation [140,232], cytokine storm syndrome [228] and autoimmune diseases [209,233], as well as in severe acute respiratory syndrome coronavirus 2 (SARS-CoV-2) [228,234]. Today, program cell deaths (PCD), particularly pyroptosis along with others, such as Necroptosis [235], Ferroptosis [236], NETosis [104,237], Parthanatos [238] and PANoptosis [133,135], have received a lot of attention from all facets of research disciplines. Fortunately, in spite of scrupulous attention to unravel the underlying molecular mechanism, the phenomenon of immunological cell death is still progressively complicated [239]. Considering the impact of cell death in microbial (viral, bacterial and fungal) and parasitic infections, PCD appears to be a double-edged sword for host and pathogen survival [240]. Therefore, tight regulation of the phenomenon is crucial for immune homeostasis. In this part of the review, we focus on the interplay of the afore-listed group of NLRs (trans-activator, inflammasome and regulatory NLRs) and other critical molecules, such as type 1 interferon (IFN-I) and inflammatory caspases (Caspase 1/4/5/11), on the initiation, execution and regulation of pyroptosis (Figure 3).

In the previous sections of this review, we have emphasized that caspase 1/4/5/11 specifically cleaved the linker between the amino-terminal gasdermin-N and carboxy-terminal gasdermin-C domains in GSDMD, which was required and sufficient for pyroptosis [109,241]. During microbial and parasitic infections, regulated cell death (RCD) [103] mediated by appropriate secretion of IFN-I [242] and activation of the inflammasome is vital for the host to cope with either foreign pathogens or tissue damage. Uncontrolled activities of these players can cause aberrant tissue damage, autoinflammatory disorders, cardiometabolic diseases, cancer and neurodegenerative diseases. IFN-I was previously recognized as a crucial molecule that is involved in the protection against viral infections [243,244]; the current paradigm shift has shown its impact on a range of microbial infections, such as parasites, fungi and bacteria. Similar to cell death, IFN-I is also a double-edged sword that exhibits context-dependent functions in relation to the intrinsic and extrinsic factors in cells [242,245]. Protective host defense of IFN-I was demonstrated in *Acinetobacter baumannii* [246], *Escherichia coli* [247], *Helicobacter pylori* [91], *Legionella pneumophila* [248] *Mycobacterium abscesssus* [249], *Plasmodium berghei* [250] and *Aspergillus* species, *A. fumigatus*, *A. nidulans* and *A. tanneri* [251]. Other reports also shown that it promotes detrimental infection outcome in bacteria, such as *Chlamydia muridarum* [252], *Mycobacterium bovis* [253], *Escherichia coli* [254], *Francisella tularensis* [255], *Haemophilus influenza* [256] and *Salmonella typhimurium*-viral [257], likewise in fungal and parasitic infections, such as Candidiasis [258], as well as in Chagas parasitic infection caused by *Trypanosome cruzi* [259]. In *Listeria monocytogenes* [260,261], *Pseudomonas aeruginosa* [262,263], *Salmonella enterica serovar Typhimurium* [264,265] and *Yersinia pestis* [266], IFN-I confers dual functions depending on the IFN-stimulated genes (ISGs) involved and the route of infection. For instance, contrary to what is obtainable during foodborne transmission of listeriosis, IFN-I promotes bacterial susceptibility during intravenous route infection by creating a growth-tolerable intracellular microenvironment [261]. Mechanistically, Frantz et al. and Pagliuso et al. recently and respectively described secRNome and RNA-binding protein (Zea) secreted by *L. monocytogenes* were sensed by RIG-I to mediate IFN-I secretion and signaling pathways [260,267], and this is explored by bacteria to its advantage.

The interplay between type I IFN and cell deaths, such as apoptosis, necroptosis and pyroptosis, is obviously evident by their interconnectivity with inflammasome activities in controlling the tissue homeostasis and host defense mechanism [61,246,268] (Figure 3). NLRP3 [269], NLRP6 [168] and NLRP12 [200] and caspase 4/5/11 inflammasomes etc., possessed upstream regulatory property on caspase 1-mediated GSDMD cleavage, canonical and noncanonical NFκB activation, as well as the IFN signaling cascades depending on the pathogen and cell type. Accordingly, inflammasome NLRs positively regulate caspase 1 activation and the maturation of pyroptotic mediating cytokines (IL1β and IL18) via ASC, except NLRC4 activation, which can proceed independently of ASC for the enzymatic processing of pro-caspase 1 without autoproteolysis and efficient cytokine processing [270]. IFN-I dependent NLRP6 inflammasome activation also induces the activation of caspase 11 along with caspase 1 activation via ASC for boisterous IL18 processing and enhanced pyroptosis mediated by caspase 1 and GSDMD [168,169] in *Listeria* and *Streptococcus Pneumoniae* infections. Pathogen recognition via TLRs, CLRs [101] and TNFR transduces signals, which may or may not go through NODosome to orchestrate nuclear translocation of NFκB, being essential for NLRP3 priming and promotes transcription of caspase 11, pro- IL1β and pro-IL18 [103,269]. Although cytosolic recognition of NOD1/2 may not be evidently linked directly to caspase 1 activation, their signaling complex influences the phosphorylation of NFκB, and this presumably elicits caspase 11 transcriptional expression. The antiviral potency of IFN-I is mediated via several receptors, such as TLRs (TLR3, TLR7 and TLR4) and cytosolic receptors (such as RIG-I, cGAS, STING and others) via TBK1 and phosphorylation of interferon regulatory factors, such as IRF3 and IRF7 [200,271]. Interestingly, recent studies revealed that regulatory NLRs, such as NLRXI, NLRC3 [272,273], NLRP12 and NLRP6, suppress NFκB and IFN-1, which mediate principal actors of cell death, as shown in Figure 3, though the link between these receptors and cell death is still unfathomable and thus requires further investigations. NLRP12 [193,194,195], NLRX1 [2,4,177,179] and NLRP6 [169,173] have been demonstrated to suppress proinflammatory cytokines chiefly orchestrated by NFκB, ERK and MAPK signaling pathways. NLRP12 also degrades NODosome to limit nuclear translocation of NFκB during bacterial infection [197]. Various studies have described NLRP12 as a critical innate immune checkpoint in cancer and pathogenic infections. The latest mechanistic study from our research group showed that NLRP12 regulates RIG-I mediated IFN-I signaling; thus, its possible expansion may encompass pyroptotic cell death, interferonopathy-based autoimmunity and associated autoinflammatory diseases based on that key rationale.

## 8. Conclusions

Microbial and parasitic infections provoke immunological responses upon their recognition by immune receptors; these include the activation of the innate immune system, the induction of systemic inflammation and cell death. Regulated cell death mediates minimal consequences; therefore, apoptosis, pyroptosis, necroptosis, ferroptosis and PANoptosis, as well as others, required tight regulation to avert uncontrolled inflammation and autoimmune conditions. The activities of NOD-like receptors play vital roles in the initiation and execution of cell death, and therefore, become crucial targets not only in mounting the regulated protective innate immunity but also as an antidote for the uncontrolled inflammatory conditions. Since December 2019, the entire world has been facing the lethal challenge of pandemic transmission of severe acute respiratory syndrome coronavirus 2 (SAR-CoV-2) critically mediated by the overproduction of proinflammatory cytokines leading to multi-organ dysfunction and virus-induced cytokine storm. Cell death and inflammatory responses have been closely linked to the pathological progression of SAR-CoV-2; thus, exploring regulatory NLRs will help in the development of a good therapeutic apparatus and also provide detailed molecular mechanisms underlying this pandemic outbreak. However, despite extensive studies on NLRs, more investigations are still outstanding, such as their impacts on drug resistance and regulation of adaptive immunity.

## Figures and Tables

**Table 2:** Structure and classification of NLRs family.

NLRs Family	Sub-Family/Domain Architectures	Gene
Acidic transcription-carrying domain (NLRA)	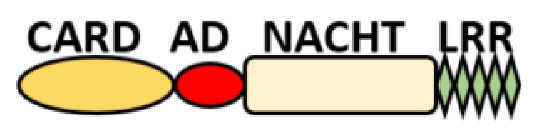	*CIITA (NLRA)*
BIR- carrying domain (NLRB)	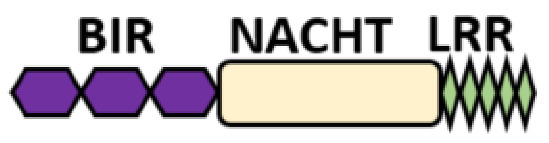	*NLRB (NAIP)*
CARD—carrying domain (NLRC)	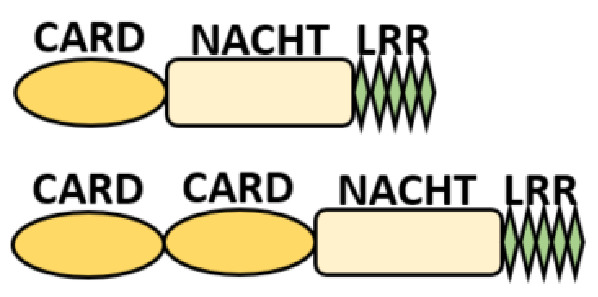	*NOD1, NLRC4* *NOD2*
PYD-carrying domain	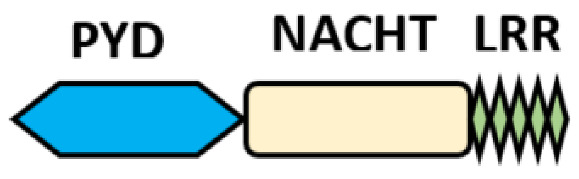	*NLRP2-NLRP9* *NLRP11-NLRP14*
	Additional domain (FIIND) 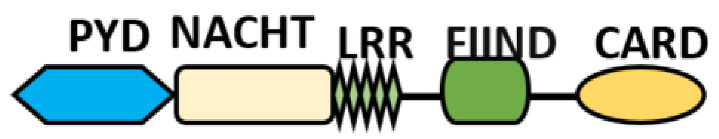	*NLRP1*
	No LRR 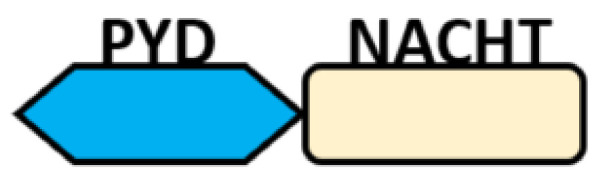	*NLRP10*
Unidentified domain	NLRs without PYD nor CARD 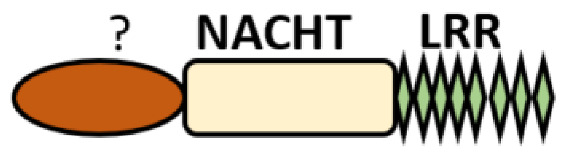	*NLRC3* *NLRC5* *NLRX1*
NLR-related molecules	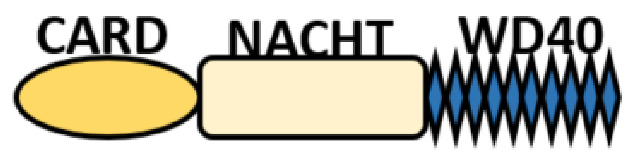	*Apaf-1*

CARDs, Caspase activation and recruitment domains; PYD, PYRIN domain; NACHT, Nucleotide-binding and oligomerization; BIR, Baculoviral inhibitory repeat; AD, Acidic domain; FIIND, Function to find; LRR, Leucine-rich repeat; WD40, Beta-transducin or WD-40 repeat.

## Data Availability

Not applicable.
